# Kynurenine derivative 3-HAA is an agonist ligand for transcription factor YY1

**DOI:** 10.1186/s13045-021-01165-4

**Published:** 2021-09-25

**Authors:** Zhaopeng Shi, Guifang Gan, Xiang Xu, Jieying Zhang, Yuan Yuan, Bo Bi, Xianfu Gao, Pengfei Xu, Wenbin Zeng, Jixi Li, Youqiong Ye, Aiwu Zhou, Naixia Zhang, Wen Liu, Shuhai Lin, Jun Mi

**Affiliations:** 1 Basic Medical Institute, Key Laboratory of Cell Differentiation and Apoptosis of the Chinese Ministry of Education, Hongqiao International Institute of Medicine, Tongren Hospital, Shanghai Jiao Tong University School of Medicine, Shanghai, China; 2 Department of Nuclear Medicine, Rui Jin Hospital, Shanghai Jiao Tong University School of Medicine, Shanghai, China; 3 Shanghai Profleader Biotech Co., Ltd, Shanghai, China; 4 Xiangya School of Pharmaceutical Sciences, Central South University, Changsha, China; 5 School of Life Science, Fudan University, Shanghai, China; 6 CAS Key Laboratory of Receptor Research, Department of Analytical Chemistry, Shanghai Institute of Materia Medica, Chinese Academy of Sciences, Shanghai, China; 7 School of Pharmaceutical Sciences, State Key Laboratory of Cellular Stress Biology, Xiamen University, Xiamen, China; 8 School of Life Sciences, State Key Laboratory of Cellular Stress Biology, Xiamen University, Xiamen, China

**Keywords:** 3-Hydroxyanthronic acid (3-HAA), Kynurenine, YY1, DUSP6, Hepatocellular carcinoma (HCC), Tryptophan metabolism

## Abstract

The 3-hydroxyanthranilic acid (3-HAA), a derivative of kynurenine, was reported to suppress tumor growth. However, the function of 3-HAA largely remains unclear. Here, we report that 3-hydroxyanthranilic acid (3-HAA) is lower in tumor cells, while adding exogenous 3-HAA induces apoptosis in hepatocellular carcinoma by binding YY1. This 3-HAA binding of YY1 leads to phosphorylation of YY1 at the Thr 398 by PKCζ, concomitantly enhances YY1 chromatin binding activity to increase expression of target genes. These findings demonstrate that 3-HAA is a ligand of YY1, suggesting it is a promising therapeutic candidate for HCC.

**Supplementary Information:**

The online version contains supplementary material available at 10.1186/s13045-021-01165-4.

## Highlights

1. 3-HAA induces apoptosis of HCC cells by binding YY1
2. 3-HAA recruits PKCζ to phosphorylate T398 of YY1

Tryptophan metabolism is enhanced in various tumors by upregulating the indoleamine 2,3-dioxygenase 1/2 (IDO1/2) or tryptophan 2,3-dioxygenase (TDO2) [1–3]. The 3-hydroxyanthranilic acid (3-HAA), a derivative of kynurenine, was reported to suppress tumor growth [4]. However, the function of 3-HAA largely remains unclear.

## 3-HAA induces apoptosis by binding with transcription factor YY1

**Fig. 1:**
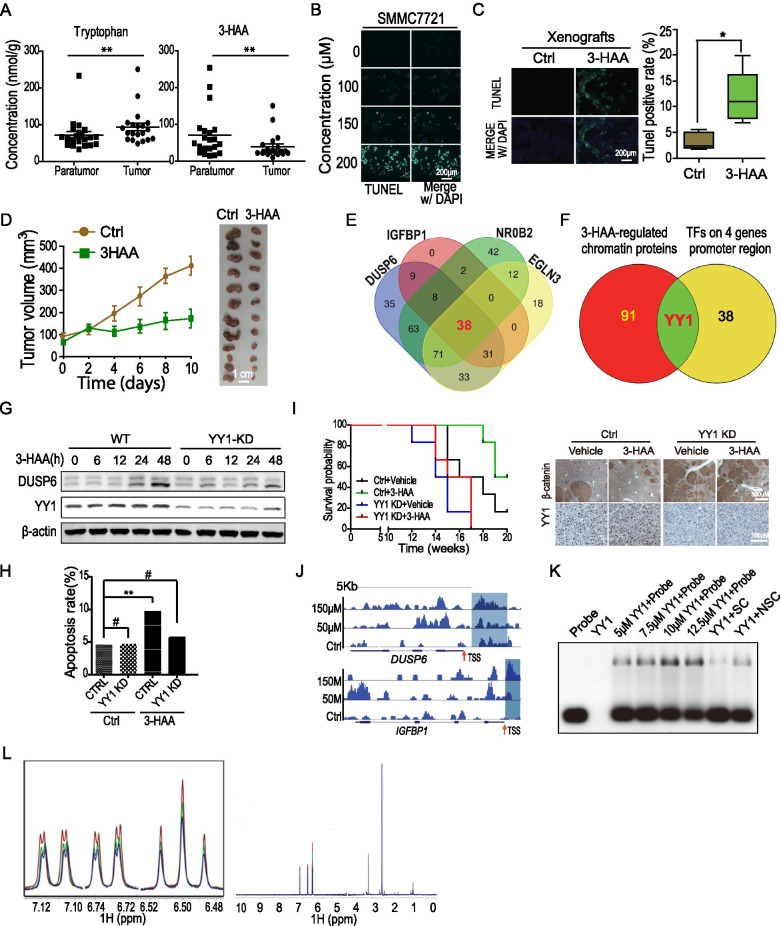
3-HAA induces apoptosis by binding with transcription factor YY1. **A** Quantitative analysis of tryptophan metabolites by LC–MS/MS and GC–MS in HCC and the corresponding paratumor tissues. **: *P* < 0.01. **B** Apoptosis analysis by TUNEL assay in SMMC7721 cells treated for 24 h with 3-HAA at the indicated dose. **: *P* < 0.01. **C** Apoptosis analysis by TUNEL assay and flow cytometry in SMMC7721 xenografts with and without 3-HAA treatment. The dose of 3-HAA was 100 mg/kg day. Mice were treated for seven days and sacrificed on day 10. *: *P* < 0.05. **D** The growth curve and representative photo of PCX xenografts. **E** The common proteins between 3-HAA-increased chromatin-binding proteins and predicted transcription factors binding to the top 4 genes’ promoter region. **F** The common proteins between 3-HAA-increased chromatin-binding proteins and predicted transcription factors binding to the promoter region of the top 4 genes. **G** YY1 knockdown abolished 3-HAA-induced *DUSP6* expression. SMMC7721 cells were treated with 100 μM of 3-HAA for the indicated time. **H** The effect of YY1 knockdown on 3-HAA-induced HCC apoptosis, analyzed by flow cytometry. The concentration of 3-HAA was 100 μM. The **: *P* < 0.01. **I** YY1 depletion attenuated 3-HAA suppression of HCC tumor formation and shorten the survival of mice bearing transposon HCCs. The transposon genetic HCC mouse model was established as described in the section of Methods and Materials (n = 6). **J** The ChIP-sequencing analysis of YY1 on the *DUSP6* and *UGFBP1* genes. The HCC cells were treated with the indicated dose of 3-HAA prior to ChIP-sequencing. **K** 3-HAA binding to YY1 was determined by electrophoretic mobility shift assay (EMSA). The synthesized oligonucleotide was labeled at the 5' terminus with FAM, and YY1 was purified using a Hitrap heparin column. **L** NMR measurement of direct binding between 3-HAA and YY1. T1r NMR spectra for PBS (red) alone or in the presence of YY1 at 5 µM (green), or 8 µM (blue). The CPMG spectrum for 6b was recorded in the presence of 5 mM YY1

Utilizing liquid chromatography-tandem mass spectrometry (LC–MS/MS), we first found that the concentration of 3-HAA decreased in 37 cases of HCC (*p* < 0.01) compared to the matched paratumor tissues (Fig. 1A). The gene ontology (GO) analysis revealed that the apoptosis pathway was highly activated in 3-HAA-treated HCC cells (Additional file 1: Fig. S1A), which was confirmed by the TUNEL assay that apoptosis was increased in a dose-dependent manner in 3-HAA-treated SMMC7721 cells and HCC xenografts (Fig. 1B, C). Levels of cleaved caspase 3 and cleaved PARP were increased in a dose- and time-dependent manner in the SMMC7721 and HepG2 cells (Additional file 1: Fig. S1B). Consequently, 3-HAA treatment led to suppression of HCC xenografts growth (Fig. 1D).

The gene expression profiles of SMMC7721 or HepG2 cells were further analyzed, and the top 21 upregulated genes at all three-time points (1, 8, or 24 h) after the start of 3-HAA treatment were selected (Additional file 1: Fig. S1C). The 38 common transcription factors were first selected from those proteins that potentially bind to the promoter region (− 5000 to + 1) of the top 4 genes [5] (Fig. 1E and Additional file 1: Fig. S1D). Through tandem mass-tagged quantitative proteomics analysis, the 91 proteins are increasingly bound to chromatin from the 1^st^ hour to the 8^th^-hour post-3-HAA treatment (Additional file 1: Fig. S1E), and YY1 was the only protein overlapped with the predicted transcription factors that potentially bound to the promoter region of the top 4 genes (Fig. 1F). The function assay showed that YY1 knockdown abolished 3-HAA-induced upregulation of target genes in SMMC7721 cells (Fig. 1G), and 3-HAA-induced apoptosis was reduced in SMMC7721 cells depleted of YY1 (Fig. 1H, Additional file 1: Figs. S1F, and S1G). Consequently, 3-HAA reduced tumor numbers and prolonged mice survival in mice with transposon-induced HCCs, whereas the same dose of 3-HAA had no remarkable effect on tumor numbers and mice survival once YY1 depleted.

The ChIP-sequencing analysis demonstrated that 3-HAA induced the union peak formation of YY1 on the promoter region of the top 4 *genes* (Fig. 1J & Additional file 1: Fig. S1I). 3-HAA increased YY1 binding to the promoter sequence of *DUSP6* in a dose-dependent manner, as evidenced by an in vitro electrophoretic mobility shift assay (Fig. 1K). Furthermore, nuclear magnetic resonance was performed to determine whether 3-HAA directly binds YY1. Dose-dependent signal attenuation was observed in the T1r NMR spectrum, suggesting that YY1 directly interacts with 3-HAA (Fig. 1L).

## PKCζ phosphorylates YY1 at Thr 398 in response to 3-HAA

**Fig. 2:**
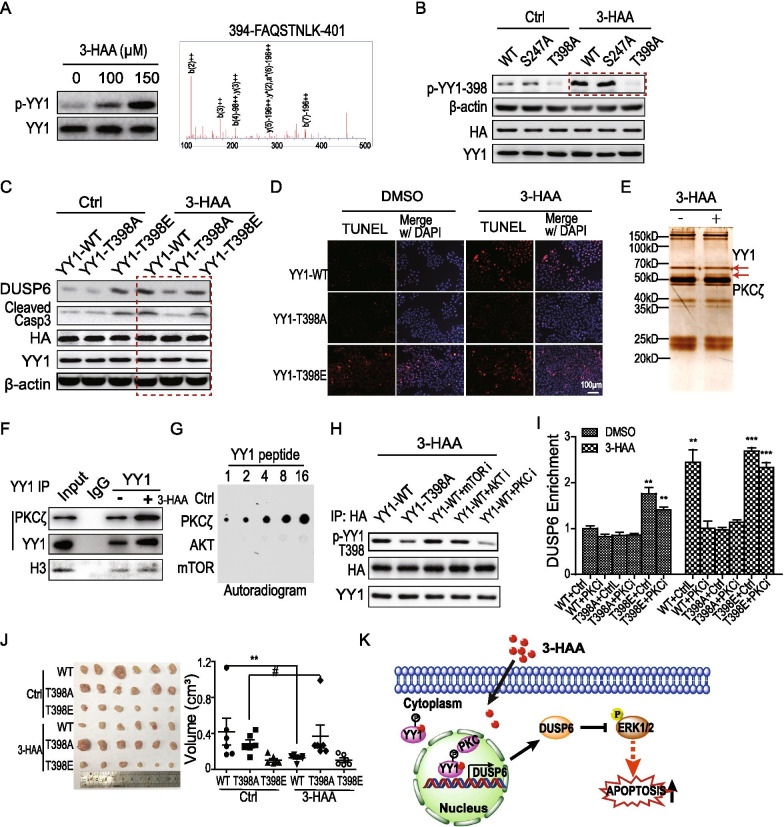
PKCζ phosphorylates YY1 at Thr 398 in response to 3-HAA. **A** The YY1 phosphorylation was analyzed by immunoblotting and mass spectrometry 2 h after 3-HAA treatment. The YY1 was blotted on the enriched phospho-proteins from SMMC7721 cells. The YY1 modification was analyzed by the mass-spectrometry following YY1 immunoprecipitation. **B** The T398A but not S247A mutation abolished 3-HAA-induced YY1 phosphorylation. The YY1 was conjugated with HA tag. The YY1 phosphorylation was detected by the T398 phospho-specific antibody. **C** The mutation of T398E in YY1 promoted DUSP6 expression and apoptosis, whereas the T398A mutation suppressed DUSP6 upregulation. The YY1 was fused with HA tag. **D** The YY1 mutation of T398A reduces the 3-HAA-induced apoptosis, analyzed by the TUNEL assay. **E** 3-HAA increased PKCζ binding to YY1, analyzed by co-immunoprecipitation. **F** 3-HAA increased PKCζ binding to YY1, analyzed by immunoblots following immunoprecipitation. **G** The kinase screening for T398 phosphorylation of YY1. The kinase candidates were predicted by online tools NetPhos 3.1 (www.cbs.dtu.dk/services/NetPhos) [8] and GPS 5.0 (gps.biocuckoo.cn) [9]. **H** The effect of kinase inhibitors on 3-HAA-induced YY1 phosphorylation. The YY1 phosphorylation was detected by the T398 phospho-specific antibody. The concentration of 3-HAA was 100 μM. AKT inhibitor, MK2206 (0.5 μM); PKC inhibitor, Go6983 (0.5 μM); mTOR inhibitor, rapamycin (0.1 μM). **I** The effect of PKC inhibitor Go6983 on the YY1 enrichment at the *DUSP6* promoter in SMMC-7721 cells. **: *P* < 0.01, ***: *P* < 0.001. **J** The T398A mutation of YY1 abolished the 3-HAA-inhibited HCC xenografts growth (n = 6). **: *P* < 0.01. **F** and **G** The apoptosis analysis in xenografts by TUNEL assay and the flow cytometry. The histogram presents as mean ± SD (*: *P* < 0.05; **: *P* < 0.01.). *Note*: The dose of 3-HAA used in **A** was 100 µM. **K** The proposed 3-HAA binding model with YY1

Thus, the YY1 phosphorylation was further analyzed to determine whether 3-HAA induced YY1 phosphorylation. The immunoblotting following phosphorylation protein enrichment showed that 3-HAA increased YY1 phosphorylation, and mass spectrometry analysis revealed that the T398 of YY1 was phosphorylated in 3-HAA-treated HCC cells (Fig. 1A). The T398A mutation diminished the 3-HAA-induced YY1 T398 phosphorylation (Fig. 2B). The function analysis displayed that the T398A mutation of YY1 suppressed 3-HAA-upregulated DUSP6 expression and reduced the level of cleaved Caspase 3/cleaved PARP, whereas the mimic phosphorylation of T398E mutation promoted DUSP6 expression even without 3-HAA treatment (Fig. 2C). The TUNEL assay and the flow cytometry analysis demonstrated that the T398A mutation of YY1 suppressed 3-HAA-induced apoptosis (Fig. 2D & Additional file 1: Fig. S2A), suggesting T398 phosphorylation of YY1 is critical for 3-HAA-induced apoptosis.

Moreover, the kinase screening assay was performed on the FAQSTNLK peptide of YY1. Proteomic analysis by mass spectrometry following YY1 immunoprecipitation showed that 3-HAA increased the association of YY1 with PKCζ, which were cosistent with the kinase prediction [6, 7] and further confirmed by immunoblotting, suggesting that 3-HAA recruits PKCζ to phosphorylate YY1 (Additional file 1: Figs. S2B, Fig. 2E, F). The kinase PKCζ significantly increased the peptide phosphorylation, reflected by the autoradiogram on the dot blot (Fig. 2G). Also, only the PKCζ inhibitor markedly decreased YY1 T398 phosphorylation (Fig. 2H), suggesting that PKCζ is the kinase for T398 phosphorylation of YY1 induced by 3-HAA.

Moreover, the T398E mutation but not T398A mutation of YY1 increase the YY1 binding on the *DUSP6* promoter, no matter with or without 3-HAA, and the PKCζ inhibitor markedly decreased the YY1 binding on *DUSP6* promoter in the SMMC7721 cells depleted of endogenous YY1 and expressing exogenous *wild type* YY1, but not in the cells expressing T398A/T398E mutant YY1 (Fig. 2I). Also, 3-HAA had no effect on tumor growth in mice with HCC xenograft depleting endogenous YY1 and expressing T398A mutant YY1. Whereas the same dose of 3-HAA significantly decreased xenograft growth expressing *wild type* YY1 (Fig. 2J). The clinical data that the PKCζ expression level was closely correlated with the overall survival of the grade I HCC patients (Additional file 1: Fig. S2C) further supported these findings.

In brief, our results have determined that 3-HAA is an active metabolite regulating tumor cell fate by binding to and activating the transcription factor YY1 (Fig. 2K). The T398 phosphorylation of YY1 promots YY1 binding to its target sequence. Exogenous 3-HAA induces tumor cell apoptosis and inhibits HCC growth, suggesting its potential use in HCC therapy.

## Supplementary Information

**Additional file 1**. Supplemental Figures & the Methods and Material.
