# Supplementary material for: Kynurenine derivative 3-HAA is an agonist ligand for transcription factor YY1
Source: J Hematol Oncol. 2021 Sep 25;14:153. doi: 10.1186/s13045-021-01165-4 (PMC8465765; doi:10.1186/s13045-021-01165-4)
Supplement: Supplementary file 1 — Additional file 1. Supplemental Figures & the Methods and Material. [file 13045_2021_1165_MOESM1_ESM.pdf]

Figure S1 3-HAA induces apoptosis in HCC cells by binding to YY1. Related to Figure 1

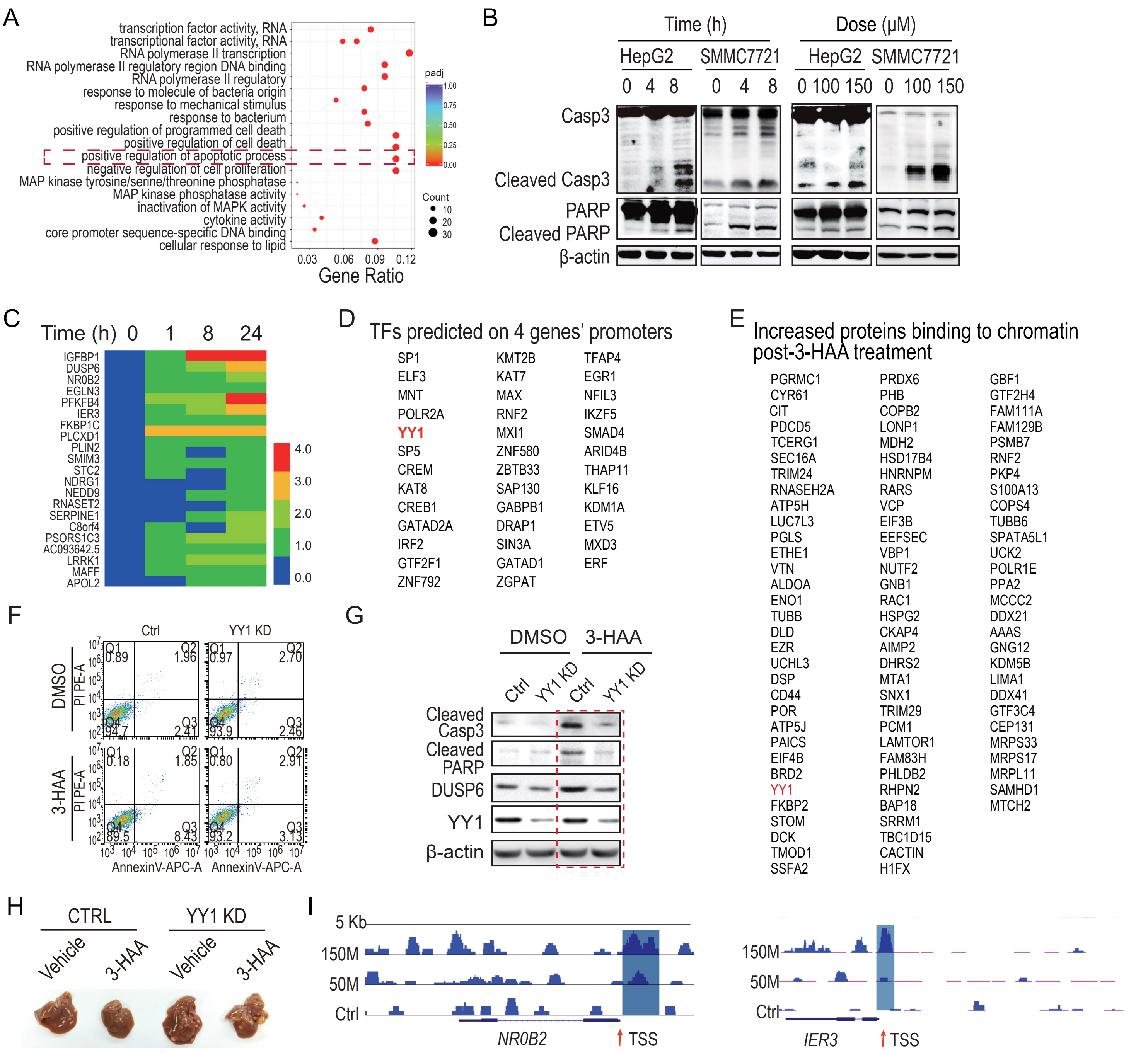

Figure S1 legend

A. Gene Ontology (GO) enrichment analysis on the expression of HCC SMMC7721 cells treated by 100 μM of 3-HAA for 8 hours.

B. Analysis of apoptotic signaling in HCC cells treated with 100 μM 3-HAA for indicated time or treated for 12 h with 3-HAA at the indicated dose.

C. The heatmap of the top consistently upregulated genes in SMMC7721, Hep3B, and SMMC97H. Deep sequencing was used to profile gene expression in HCC SMMC7721, Hep3B, and SMMC97H cells. HCC cells were treated with 100 μM 3-HAA for 1, 8, or 24 h.

D. The common transcription factors potentially binding to the promoter region (-5000 to +1) of top 4 genes, which was up-regulated in 3-HAA-treated SMMC7721 cells.

E. Proteins increasely binding to chromatin after 3-HAA treatment, analyzed by quantitative proteomics analysis.

F. The apoptosis analysis by flow cytometry in SMMC7721 cells depleted of YY1 and/or overexpressing DUSP6.

H. The representative transposon-induced tumors in each group.

G. The effect of YY1 knockdown on 3-HAA-activated apoptotic signaling. The concentration of 3-HAA was 100 μM.

I. The peak of ChIP-Seq on the promoter of 3 representative upregulated genes.

Figure S2 PKCζ phosphorylates YY1 at Thr398 in response to 3-HAA. Related to Figure2

A

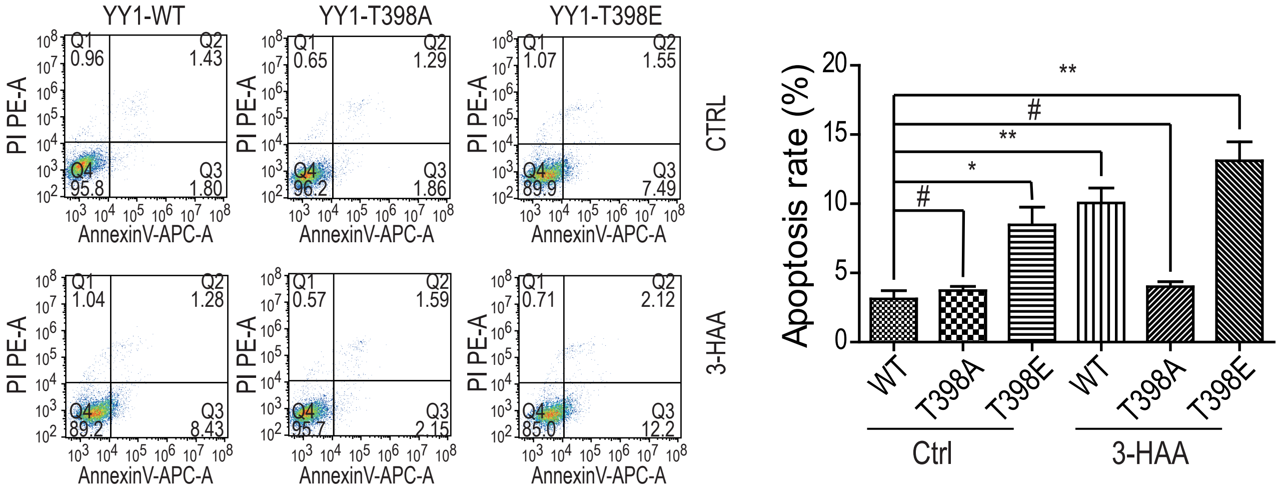

B

YY1 binding protein	Control	3-HAA
PPIB	✓	✓
FLG2	✓	
LYZ	✓	✓
HSPA5	✓	✓
HSPA9	✓	✓
HSPD1	✓	✓
Exportin 1	✓	
HRNR		✓
HNRNPA2B1		✓
SLC25A4		✓
PDIA6		✓
HNRNPA0		✓
RuvBL1	✓	✓
RuvBL2	✓	✓
PKCζ		✓

C

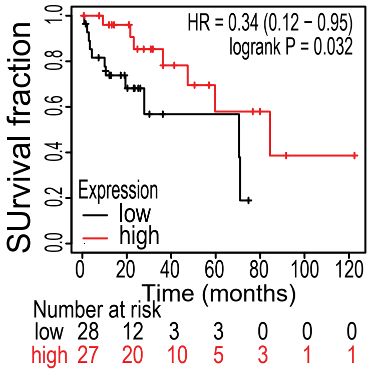

Figure S2 legend

A. The apoptotic SMMC7721 cells determined by the flow cytometry analysis. The concentration of 3-HAA was 100 μM.

B. The representative YY1 binding proteins identified by protein mass-spect analysis following co-immunoprecipitation.

C. The effect of PKCζ expression on grade 1 liver cancer overall survival. The total patient number was 55 and devided into two group by the mean, $p < 0.05$.

Methods and Materials

Cells

Human HCC cell line HepG2 (American Type Culture Collection; Manassas, VA, USA; RRID: CVCL_0027), Hep3B (RRID: CVCL_0326), PLC8024 (RRID: CVCL_0485) (Cell Bank of the Chinese Academy of Science, Shanghai, China), MHCC97L (RRID: CVCL_4973), MHCC97H (RRID: CVCL_4972), Huh7 (RRID: CVCL_0336), LO2 (RRID: CVCL_6926), WRL68(RRID: CVCL_0581) and SMMC7721 (RRID: CVCL_0336) (Genechem Co., Ltd., Shanghai, China) were grown at 37 °C in DMEM (Invitrogen, Grand Island, NY, USA) containing 5% CO_2 atmosphere and supplemented with 10% heat-inactivated fetal bovine serum (PAA, Australia) in the presence of 100 U/mL penicillin and 0.1 mg/mL streptomycin.

Plasmids

The pGIPZ-shHAAO-1, pGIPZ-shHAAO-2, pGIPZ-shHAAO-3, pGIPZ-shDUSP6-1, pGIPZ-shDUSP6-2, pGIPZ-shYY1-1 and pGIPZ-shYY1-2 were purchased from SHSMU DNA Library. The pTSB-KMO was purchased from Genewiz. pLenti-HA-YY1-WT was constructed by PCR using primers WT-F: CCGGAATTCATGTACCCATACGACGTCCCAGACTACGCTGCCTCGGGCGACAC and WT-R: CGCGGATCCTCACTGGTTGTTTTTGGCCTTAGC. The YY1-T398A and YY1-T398E mutant vectors are obtained using mutagenesis kit (TOYOBO, cat: SMK-101) and primers YY1-F-T398E: gtttgctcagtcaGAAaacctgaaatctcacatc, YY1-F-T398A: gtttgctcagtcaGCAaacctgaaatctcacatc and YY1-R-mut: ttcttattacaaccatcgaaggggcacac. For the construction of pET-28a(+)-YY1, a BamHI/EcoRI fragment encompassing the CDS of human YY1 (NM_003403.4) was amplified from human cDNA by PCR using primers YY1-F: CGGGATCCATGGCCTCGGGCGACACCC and YY1-R: CCGGAATTCTCACTGGT TGTTTTTGGCCTTAGCA, and the fragment was inserted into pET28a(+) vector for bacterial expression.

Colony formation

HCC cells were seeded into 6-well dishes at a cell density of 1000 cells/well and treated with drugs for 10-14 days until clones were visible. PBS-washed cells were fixed with 4% paraformaldehyde and stained with 1% crystal violet. The stained clones were counted.

Cell proliferation assay

Cell proliferation was measured using the Cell Counting Kit-8 reagent (CCK-8, Dojindo, cat: CK04). HCC cells were seeded into a 96-well plate at 2000 cells/well, were treated with 3-HAA (Sigma, cat: 148776) and ZVAD (TargetMol, cat: T6013) at appropriate doses as indicated in the figure legend. CCK-8 assays were performed in triplicates as instructed by the manufacturer for 2 hours at indicated time points. Absorbance was measured at 450 nm using a microplate reader, and cell viability was normalized to control, and the mean of at least three independent experiments was calculated.

Flow cytometry

HCC cells were treated with 3-HAA at appropriate doses for 48 hours and harvested by trypsinization and washed with phosphate-buffered saline (PBS). The cells were then stained with anti-human Annexin V-APC and PI-PE (Multisciences Inc, cat: AP107-100) for 30 min. At least 1 x 10^6 cells were analyzed by a FACS Aria II (BD Falcon, Franklin Lakes, NJ, USA). Cells were gated based on their forward and side scatter properties. Furthermore, mouse xenograft tissues were minced and digested with collagenase and DNase Ⅰ (Transgen, GD201-01) at 37 °C for 30 min and then filtered with 40-micron cell strainers (BD Falcon). Tumor cells were stained with annexin-V/propidium and analyzed by flow cytometry.

ChIP analysis

Chromatin was isolated from HCC cells treated with or without 3-HAA and fragmented to a size range from 150 to 400 bp. The solubilized chromatin fragments were immunoprecipitated

with antibodies against YY1 (Active Motif, cat: 61779). The recovered DNA fragments were processed for DNA sequencing by the Illumina Genome Analyzer. The generated short reads were mapped onto the genome, and the peak calling program was used to identify peaks with the mapped reads.

For ChIP-PCR and ChIP-QPCR, primers (ChIP-NC-F: CACCTGCTTAGCACAGTTTCCA, ChIP-NC-R: GTCCAGCAAAACCTGATGGATT, ChIP-DUSP6-F: CCTCCATCCGGCTTCCAAT, ChIP-DUSP6-R: GGTAACTTTGGGGAGGTGCG, ChIP-PC-F: GACAGGTCTGAAGCCTGGAG, ChIP-PC-R: CGGGACGTGAAAGGTTAGAA) were used to detect the accumulation of YY1 in the DUSP6 promoter.

Western blotting assays

Appropriate cells were lyzed in RIPA lysis buffer containing a cocktail of protease inhibitors (Roche) and PMSF. Total protein concentration was determined using the bicinchoninic acid (BCA) assay kit (Ding Guo Biotechnology, cat: BCA02). For nuclear and cytoplasmic protein analysis, the Nuclear and Cytoplasmic Protein Extraction Kit (Beyotime, cat: P0028) was used according to the instructions. For immunoprecipitation, HCC cells were lyzed in lysis buffer (50mM Tris, 150mM NaCl, 1% TritonX-100, and 1mM EDTA) containing a cocktail of protease inhibitors and PMSF. Protein A/G beads, HA/YY1 antibodies were added to soluble protein and incubated overnight at 4°C, with gentle agitation. Immunoprecipitated materials were washed three times, eluted with loading buffer at 95°C for 5 min, and analyzed by western blotting. Antibodies against the following proteins were used for immunoblotting: IDO1 (CST, cat: 86630), TDO2 (Origene, cat: TA504879), KYNU (Proteintech, cat: 11796-1-AP), KMO (Proteintech, cat: 10698-1-AP), HAAO (Proteintech, cat: 12791-1-AP), Cleaved Caspase3 (CST, cat:9315), PARP (Proteintech, cat: 13371-1-AP), DUSP6 (Proteintech, cat: 10433-1-AP), IGFBP1 (Proteintech, cat: 13981-1-AP), p-ERK (CST, cat: 4370), ERK (CST, cat: 4695), BAD (CST, cat: 9239), BCL2 (CST, cat: 4223), BCL-XL (CST, cat: 2764), YY1 (Active Motif, cat:

61779), H3 (Proteintech, cat: 17168-1-AP), p-YY1 (Abclonal, cat: E13449), HA (Sangon, cat: D110004), PKCζ (CST, cat: 9368), RIP3 (Proteintech, cat: 17563-1-AP), P62 (CST, cat: 5114), LC3B (CST, cat: 2775), CYP1A1 (Proteintech, cat: 13241-1-AP), CYP1B1 (Proteintech, cat: 18505-1-AP), TIPARP (Sigma, cat:SAB2102431) and β-actin (Santa Cruz, cat: 47778). The immunoblots were scanned using an Odyssey infrared imaging system (LI-COR). Immunolabeling was detected using the ECL reagent (Sigma). Protein expression was normalized against β-actin.

Real-time quantitative PCR

Total cellular RNA was prepared using the TRIzol reagent (Invitrogen, cat: 15596018) as instructed by the manufacturer and was reverse transcribed using a reverse transcription reagent kit (TAKARA, cat: RR036A). After cDNA synthesis, real-time quantitative polymerase chain reaction (PCR) was performed in triplicate in a 96-well plate with an ABI7500 real-time PCR system (Life Technologies, Grand Island, NY, USA) using SYBR Green mixture (AG, cat: AG11702). CYP1A1, CYP1B1, and TIPARP expression were normalized against β-actin. The primer sequences were as follows: *IGFBP1*-F 5'- GGCTCTCCATGTCACCAACA -3', *IGFBP1*-R 5'- CCATTCCAAGGGTAGACGCA -3', *DUSP6*-F 5'- CCTGCATTGCGAGACCAATC -3', *DUSP6*-R 5'- GGGGGTGACGTTCAAGATGT -3', *NR0B2*-F 5'- GCCTGAAAGGGACCATCCTC -3', *NR0B2*-R 5'- CCAGGGTTCCAGGACTTCAC -3', *EGLN3*-F 5'- CTTTGTGGCCTTCTTTGA AGT -3', *EGLN3*-R 5'- CCACACAGTTGCTCCACAT -3', *PFKFB4*-F 5'- GGATCCCGGACCTCGATTCT -3', *PFKFB4*-R 5'- CCCAGGAAGTTGTCCAGGTAG -3', *IER3*-F 5'- AAGCCCATCCACCGCTAAAA -3', *IER3*-R 5'- AGAAGCCTTTTGGCTGGGTT -3', *CYP1A1*-F 5'- TCGGCCACGGAGTTTCTTC -3', *CYP1A1*-R 5'- GGTCAGCATGTGCCCAATCA-3', *CYP1B1*-F 5'- TGAGTGCCGTGTGTTTCGG-3', *CYP1B1*-R 5'- GTTGCTGAAGTTGCGGTTGAG-3', *TIPARP*-F 5'-

AGAACGAGTGGTTCCAATCCA-3’, *TIPARP-R* 5’- TGGGTGCAAAAGATCAGTCTG-3’, *β-actin*-F 5’-GCGGGAAATCGTGCGTGACATT-3’ and *β-actin*-R 5’-GATGGAGTTGAAGGTAGTTTCG-3’.

Immunohistochemistry

Tissue samples were fixed in 4% paraformaldehyde (PFA) and embedded in paraffin. Primary antibodies used were 3-HAA (Abcam, ab15580), KYNU (Proteintech, cat: 11796-1-AP), KMO (Proteintech, cat: 10698-1-AP), HAAO (Proteintech, cat: 12791-1-AP), IDO1 (CST, cat: 86630), and DUSP6 (Proteintech, cat: 10433-1-AP). Detection was performed with the Elivision super Kit (MXB, cat: KIT-9921) and DAB Substrate (Boster, cat: AR1022), followed by hematoxylin counterstaining (BBI, cat: E607317).

Expression and Purification of HIS-YY1 Fusion Protein

A plasmid containing N-terminal 6×His-tagged human YY1 coding sequence was transformed into BL21 (DE3) cells. A new bacterial colony was inoculated into LB media containing Kanamycin and grown overnight at 37°C. These bacteria were diluted 1:100 in 500 mL pre-warmed LB with Kanamycin and grown for 3 hours at 37°C. After induction of YY1 expression with 1mM IPTG at 16℃ for 16 hours, cells were collected. Pellets were resuspended in Buffer A (20mM imidazole, 20mM Tris, 300mM NaCl, pH7.4) and disrupted by a high-pressure cell breaker. After separating the debris by centrifugation, the supernatant was loaded on a Ni column preequilibrated with Buffer A. The His-YY1-tagged protein was eluted by a 0.02–0.2 M imidazole gradient with Buffer B (300 mM NaCl, 20 mM Tris and 200 mM imidazole pH 7.4). The peak fractions were collected and dialyzed against Buffer C (10mM Tris and 100mM NaCl). Subsequently, proteins were loaded onto a Hitrap heparin column and eluted by a NaCl gradient (0.1–1.5 M). The peak fractions were collected and dialyzed against PBS buffer. Proteins were then concentrated for use.

Electrophoretic mobility shift assay

Promoter fragments were generated by primers (DUSP6-1145-1134-F: ACGCCTCCATCCGGCTTCCA, DUSP-1145-1134-R: TGGAAGCCGGATGGAGGCGT, DUSP6-1145-1134-F-mut: ACGCCTCCATCCGGCTTCCA, DUSP-1145-1134-R-mut: TGGAAGCCGGATGGAGGCGT), before labeling at 5' terminal of the primers using FAM. Promoter fragments were incubated with YY1 in ASSAY BUFFER (Tris-HCl 20mM, NaCl 100mM, $MgCl_2$ 1mM, DTT 1mM and Glycerol 4%, pH=8.0) for 20min at 37℃, and separated by polyacrylamide gel electrophoresis.

Dual-luciferase reporter

For luciferase assay, the promoter region of human DUSP6 was moved from pGL2-DUSP6 (a gift from Dr. Norton, University of Florida) by digestion, gel purified, and inserted into a KpnI/XhoI digested pGL4 vector. We constructed some mutants using primers (DUSP6-promoter-F: GGGGTACCCGAACACGCTCCTCCAGG, DUSP6-promoter-R: CCCTCGAGCGGCCGCCAGTGTGAT, DUSP6-F1-m-1598-1587: TGGCAACCTAGGCCTCGCAAAATTTCCCGGGTAGCCTACATTTCCCAAACCAG, DUSP6-R1-m-1598-1587: GGTTTGGGAAATGTAGGCTACCCGGGAAATTTTGCGAGGCCTAGGTTGCCA, DUSP6-F1-m-1145-1134: GGCCCCCACCCGGCCACGCCCTTCCTTTTCCCCAATCCGTCCGCCCCGCGG and DUSP6-R1-m-1145-1134: GCCGCGGGGCGGACGGATTGGGGAAAAGGAAGGGCGTGGCCGGGTGGGGGCC). 293T cells were co-transfected with 100ng different pGL4-DUSP6-promoter vectors, 800ng pSG5-YY1, or pSG5, and 1 ng pRL-TK-Renilla luciferase plasmids. After 48 hours, the cells were lysed, and luciferase activity was measured using the Dual-Luciferase Reporter Assay System (Promega) according to the manufacturer's instructions.

RNA-Seq

RNA was extracted from HCC cells with the TRIzol reagent (Invitrogen, cat: 15596018) according to the instructions. RNA-seq libraries were prepared with the Ovation RNASeq Systems 1-16 (Nugen), and indexed libraries were multiplexed in a single flow cell and underwent 75 base pair single-end sequencing on an Illumina NextSeq500 using the High Output kit v2 (75 cycles) at BGI Group.

Lentivirus Production

293T cells were cultured in DMEM supplemented with 10% fetal bovine serum (Gibco) and were maintained at 37°C in a humidified atmosphere with 5% CO_2. For virus production, 8 μg of the appropriate plasmid and 3.2 mg of helper plasmids (2μg pMD2.G and 6μg psPAX2) were transfected into 293T cells cultured at 80% confluence in a 10 cm dish using Lipoplus (SAGE) according to the manufacturer's instructions. Viral supernatants were collected 48 hours after transfection and filtered through a 0.45 mm filter.

Silver Stain

HCC cells were treated with 100μM of 3-HAA for 24 hours. Cells were collected and lysed by lysis buffer. Proteins were separated by SDS-PAGE and stained by using Fast Silver Stain Kit (Beyotime, cat: P0017S) according to the instructions.

Metabolism flux analysis by LC-MS/MS

For the flux experiment of tryptophan catabolism, tryptophan in the medium was replaced by fully ^{13}C-labeled tryptophan (tryptophan-$^{13}C_{11}$). Sample preparation and LC-MS/MS analysis were the same as for quantitation of tryptophan catabolic products, except that the parameters of MRM transitions were different. MRM transitions for catabolic products of tryptophan-$^{13}C_{11}$ (M11: 320 > 273) were set as 5-hydroxyindoleacetic acid (M10: 323 > 155), kynurenine (M10: 427 > 122), kynurenic acid (M10: 304 > 105), 3-hydroxykynurenine (M10: 547 > 05), xanthurenic acid (M10: 424 > 05), 3-hydroxyanthranilic acid (M7: 369 > 247), cinnabarinic acid (M14: 419 > 105). Simultaneously, the MRM transitions (M0) of unlabeled tryptophan and its catabolic products were acquired using the same settings as in quantitation experiments.

Quantitative proteomics

Cell samples were sonicated three times on ice using a high-intensity ultrasonic processor (Scientz, Ninbo, Zhejiang, China) in lysis buffer (8 M urea, 1% Protease Inhibitor Cocktail). The supernatant was collected, and proteins were reduced with five mM dithiothreitol for 30 min at 56 °C, then alkylated with 11 mM iodoacetamide for 15 min at room temperature in darkness. Following the addition of 100 mM TEAB to dilute the urea to < 2 M, trypsin was added to the protein samples first at a trypsin-to-protein mass ratio of 1:50 for digestion overnight, then at a ratio of 1:100 for second digestion lasting four h.

After trypsin digestion, peptides were desalted on a Strata X C18 SPE column (Phenomenex) and vacuum-dried. Peptides were reconstituted in 0.5 M TEAB and processed according to the manufacturer's protocol for the TMT kit/iTRAQ kit. The tryptic peptides dissolved in 0.1% formic acid (solvent A) were directly loaded onto a custom-made reverse-phase analytical column (15-cm length, 75 μm i.d.) on an EASY-nLC 1000 UPLC system. The gradient to solvent B (0.1% formic acid in 98% acetonitrile) increased from 6% to 23% over 26 min, then from 23% to 35% in 8 min and then to 80% in 3 min, after which it remained at 80% for 3 min. The flow rate was constant at 400 nL/min.

Peptides were subjected to NSI source followed by tandem mass spectrometry (MS/MS) in Orbitrap Fusion™ Tribrid™ (Thermo, CA, USA) coupled online to the UPLC. Intact peptides were detected in the Orbitrap at a resolution of 60,000. Peptides were selected for MS/MS by NCE at 35; ion fragments were detected in the Orbitrap at a resolution of 30,000. A data-dependent procedure that alternated between one MS scan followed by 10 MS/MS scans was applied for the top 10 precursor ions above a threshold intensity, which were greater than 5×10^3 in the MS survey scan with dynamic exclusion of 30.0 s. The electrospray voltage applied was 2.0 kV. Automatic gain control was used to prevent the orbitrap from overfilling; 5×10^4 ions were accumulated to generate MS/MS spectra. For MS scans, the *m/z* scan range was from 350 to 1550. The fixed first mass was set as 100 *m/z*. MS/MS data were processed using the Maxquant

search engine (version 1.5.2.8). Carbamidomethyl on cysteine was specified as a fixed modification, while oxidation on methionine was specified as a variable modification. FDR was adjusted to < 1% and the minimum score for peptides was set to > 40.

NMR detection of 3-HAA-YY1 interaction

NMR experiments based on the Carr-Purcell-Meiboom-Gill (CPMG) sequence and saturation transfer difference (STD) were applied to investigate 3-HAA-YY1 interactions. All NMR spectra were acquired at 25 °C on a 600 MHz Bruker Avance III spectrometer equipped with a cryogenically cooled probe (Bruker Biospin, Germany). Samples containing 200 μM 3-HAA and 200 μM 3-HAA in the presence of 5 μM protein were dissolved in Tris-HCl buffer (50 mM Tris-HCl, pH 7.4, 100 mM NaCl, 5% DMSO, 95% D_2O) and then used in NMR data acquisition. CPMGs were recorded using the pulse sequence of solvent-suppressed 1D ^1H CPMG (cpmgPr1d). The 90° pulse length was adjusted to approximately 11.82 μs. A total of 4 dummy scans and 64 free induction decays (FIDs) were collected into 13 K acquisition points, covering a spectral width of 8 kHz (13.3 ppm) and giving an acquisition time of 3 s. STD data were acquired using four dummy scans and a relaxation delay of 3 s, followed by 40-dB pulsed irradiation at a frequency of -1.0 ppm or alternatively 33 ppm. The total time to acquire the STD spectrum was 21 min with 128 FIDs.

Surface plasmon resonance and microscale thermophoresis

In order to measure the affinity of 3-HAA to YY1 protein, the dissociation constants were measured using a BIAcore T200 instrument (GE Healthcare, CA, USA) with a CM5 sensor chip (GE Healthcare). The YY1 protein was immobilized on a CM5 sensor chip in sodium acetate buffer (1 μg/mL, pH 5.5), and 3-HAA was gradually titrated at the indicated concentrations. The 3-HAA was injected at a flow rate of 30 μL/min. The association time was 120 s and the dissociation time was 420 s. The binding constant was calculated using a 1:1 Langmuir binding model via the BIAevaluation software.

The dissociation constant of 3-HAA for YY1 was also measured by microscale thermophoresis assay according to manufacturer's protocol (Nanotemper Technologies Ltd., Shanghai, China). YY1 protein was labeled using NT650-NHS labeling kit (Nanotemper Technologies Ltd., Shanghai, China), and the concentration of labeled YY1 protein was set to 50 nM. The unlabelled 3-HAA was gradually diluted from 2500 μM to 76.3 nM as indicated. YY1 and 3-HAA interacted in PBS buffer containing 2.5% DMSO, 0.05% Tween-20, 85mM NaCl. Measurements were made using the Monolith NT.115 (NanoTemper), and data were analyzed using MO. Affinity analysis (×64) software. Graphs were plotted using Prism 5 software.

TUNEL assay

The cover glass was placed in the 24-well plate, and the HCC cells were inoculated on the cover glass overnight. DMSO or 100 μM 3-HAA was added to the culture medium for 48 hours. The cells were washed with PBS for three times. Add 0.5mL of 4% paraformaldehyde and fix cells at room temperature for 10 minutes. Cells were treated with 0.4%Triton X-100 for 5 minutes and washed with PBS. Cells on the cover glass were treated with TUNEL staining solution and incubated in a wet box at 37℃ for 1 hour. DAPI staining solution was used to stain the nuclear for 5 minutes in dark. Cells were observed and photographed under a fluorescence microscope.

HCC-PCX mouse model

Six-week-old male BALB/c nude mice or male immune-competent C57BL/6 mice were purchased from Lingchang, Shanghai, China. Xenograft mouse model of HCC was generated by injecting SMMC-7721 HCC cells (1.5×10^6) or mouse live cancer Hep1-6 cells (1 × 10^6) subcutaneously into the armpit of the rear limb. These SMMC-7721 cells were overexpressing KMO or T398E/T398A mutant YY1 or depleted of HAAO, DUSP6, or YY1. Mice were injected intraperitoneally with 100 mg/kg of 3-HAA or an equal amount of DMSO every day. The PKC inhibitor Go6983 was injected intravenously every two days. The dose of Go6983 was 0.8

mg/kg.day, and the IDO1 inhibitor Epacadostat was orally administered at the dose of 100 mg/kg.day.

After two weeks, subcutaneously transplanted tumors were removed, and the volume was measured, and the tumors were photographed. Following homogenization or tissue slicing, the flow cytometry analysis and TUNEL assay were performed to determine the ratio of apoptotic cells in xenografts. For flow cytometry analysis, cells were collected in binding buffer and stained with Annexin V-APC and PI, and apoptotic signals were detected by flow cytometry.

HCC-PDX mouse models

This study received ethics board approval at the Shanghai Jiao Tong University School of Medicine. The HCC-PDX models (LIV#031, #046, and #057) were initially isolated from patients and were stored in liquid nitrogen. Mice were maintained under specific-pathogen-free (SPF) conditions. Once the recovered tumors grew to 250 mm^3 in mice, tumor tissues were cut into 2×2 mm pieces and implanted subcutaneously into SCID mice [23]. The 3-HAA were intraperitoneally administered every day when the tumor volume reached approximately 200 mm^3. Tumor size and mice body weight were monitored for up to 4 weeks, and tumor volume (TV) was calculated.

Transposon HCC mouse Model

This induced HCC mouse model was adopted from the literature [24-26]. Briefly, HCC inducing oncogenes β-Catenin and MET in pT2 vector along with Sleeping Beauty transposon (SB100) was introduced with GFP, pT2-shDUSP6, or pT2-shYY1 (also in pT2 vector) into C57BL/6 mice. Thirty micrograms of the oncogene plasmids and three micrograms SB100 plasmids were diluted in 2 ml of a filtered 0.9% NaCl solution and followed by an injection into the lateral tail veins of 6-week-old mice. Livers of some mice were harvested to determine tumor burden at a specific time after hydrodynamic transfection (HDT). The six mice in each group were used for survival analysis.

Statistical analysis

Data were presented as means ± SD. All data were representative of at least three independent experiments. The unpaired two-tailed Student's *t*-test and the Two-way ANOVA were used as indicated. All presented differences were $P < 0.05$ unless otherwise stated.
